# Welcome to Eduardo Cazap, Editor-in-Chief, *e*cancer

**DOI:** 10.3332/ecancer.2019.ed89

**Published:** 2019-04-01

**Authors:** Gordon McVie

**Affiliations:** 1 ecancer, 13 King Square Avenue, Bristol, BS2 8HU, UK; 2 Clinical consultant, Institute of Molecular Oncology (IFOM), Milan, Italy; 3 Cancer Studies, King’s College London, UK

The rebirth of *e*cancer.org within the new charity *e*cancer Global Foundation is a timely moment for Eduardo Cazap, the distinguished medical oncologist from Argentina, to take control of the journal. And few folk are as global as he, having been president of UICC, among many other prestigious appointments!

The new emphasis of *e*cancer.org is on cancer research in and from emerging countries, who sadly are realising that cancer is no longer a disease of rich nations. One death every 8 minutes [1] from cancer of the cervix in India, and the dominance of hepatoma in the cancer incidence tables in Africa are not new observations, but breast, prostate, lung and colorectal malignancies are steadily climbing, unchallenged by national cancer plans, in low- and middle-income countries [2]. So an expert in the field like Eduardo will do a great job leading *e*cancer through the next decade. Welcome from the *e*cancer team, who have brought about the success of our journal, and who are keen to work with you in the coming years.

‘Knowledge should be free’ was the overriding slogan which Umberto Veronesi, my co-founder, and I adopted when we set up the charity the *e*cancermedicalscience Foundation as publisher of the first ever open access peer reviewed online cancer journal with no publication charges. *e*cancer has defied the predictions that knowledge could never be free and the first threatening lawyer’s letter from a multimillion dollar medical publisher was the icing on the cake! Sadly, Umberto didn’t live to share in the tenth anniversary but would have been justly proud of its achievements. These include 50,000 downloads from PubMed Central each month, 21,000 members, an editorial board of 138 distinguished scientists and clinicians, the largest library of Key Opinion Leader oncology video interviews in the world, and around 20 million visits in 12 years from 101 countries.

Education is germane to *e*cancer, with its collection of free open access online educational courses addressing all healthcare professionals and covering old and new technologies, from palliative care in Sub-Saharan Africa, to HPV vaccination in India and immunooncology in Europe. These multi-lingual video modules help improve patient outcomes and we have evidence that they have been extremely effective. The UICC asked us to prepare and publish their universal TNM classification for the top cancers, to facilitate easy access of clinicians and nurses on a daily basis, and this too has been widely applauded.

But patients need educating too, and *e*cancerpatient has just reached puberty, with thousands of visits to watch interviews from experts interspersed with explanations of words camouflaged by ‘doctor-speak’. Education should be ‘blended’ according to the evidence, so *e*cancer has run teaching ‘face-to-face’ meetings in several continents, and most unusually on 11 occasions in Latin America, in Spanish.

There has never been a more exciting time for cancer medicine in my 50 years as a doctor, so there is a massive need for accessible free education on the new technologies, from immunoncology to protons, to pathology and imaging biomarkers, and organ sparing robotic surgery. I shall be on the touchline watching and advising the outstanding *e*cancer team, and look forward to the leadership of Eduardo Cazap.
